# Nationwide Data Support Centralised Decision-making in Penile Cancer Care: A Before-and-After Study on Guideline Adherence and Disease-specific survival for Patients with an Indication for Perioperative Oncological Treatment

**DOI:** 10.1016/j.euros.2023.03.005

**Published:** 2023-03-31

**Authors:** Emma Ulvskog, Erik K. Persson, Peter Kirrander, Katja Fall, Johan Ahlgren

**Affiliations:** a Department of Oncology, Faculty of Medicine and Health, Örebro University, Örebro, Sweden; b Regional Cancer Centre Mellansverige, Uppsala, Sweden; c Clinical Epidemiology and Biostatistics, School of Medical Sciences, Faculty of Medicine and Health, Örebro University, Örebro, Sweden; d Integrative Epidemiology, Institute of Environmental Medicine, Karolinska Institutet, Stockholm, Sweden; e Faculty of Medicine and Health, Örebro University, Örebro, Sweden

**Keywords:** Penile cancer, Chemotherapy, Radiotherapy

## Abstract

**Background:**

The benefit of perioperative oncological treatment in men with penile cancer is uncertain. In 2015, treatment recommendations were centralised in Sweden and treatment guidelines were updated.

**Objective:**

To evaluate if the use of oncological treatment in men with penile cancer increased after the introduction of centralised recommendations, and whether such therapy is associated with better survival.

**Design, setting, and participants:**

This was a retrospective cohort study including a total of 426 men diagnosed with penile cancer with lymph node or distant metastases in Sweden during 2000–2018.

**Outcome measurements and statistical analysis:**

We first assessed the change in the proportion of patients with an indication for perioperative oncological treatment who actually received such treatment. Second, we used Cox regression to calculate adjusted hazard ratios (HRs) and 95% confidence intervals (CIs) for disease-specific mortality associated with perioperative treatment. Comparisons were made for both all men without perioperative treatment and for those who did not receive treatment but who lacked apparent contraindications for treatment.

**Results and limitations:**

The use of perioperative oncological treatment increased from 2000 to 2018, from 32% of patients with an indication for treatment during the first 4 yr to 63% during the last 4 yr. In comparison to patients potentially eligible for oncological treatment who did not receive it, those who were treated had a 37% lower risk of disease-specific death (HR 0.63, 95% CI 0.40–0.98). Stage migration because of improvements in diagnostic tools over time may have inflated the more recent survival estimates. An influence of residual confounding due to comorbidity and other potential confounders cannot be excluded.

**Conclusions:**

The use of perioperative oncological treatment increased after the centralisation of penile cancer care in Sweden. Although the observational study design precludes causal inference, the findings suggest that perioperative treatment in patients with penile cancer eligible for treatment may be associated with better survival.

**Patient summary:**

In this study, we looked at the use of chemotherapy and radiotherapy for men with penile cancer and lymph node metastases in Sweden during 2000–2018. We found an increase in the use of cancer therapy and an increase in survival for patients who received such therapy.

## Take Home Message

We report on increasing use of perioperative oncological therapy for men with penile cancer in a national cohort over a 19-yr period. We found a statistically significant survival benefit in men receiving perioperative treatment, but causality cannot be proven.

## 1 Introduction

Penile cancer is an uncommon disease. The presence and extent of lymph node metastases are the most important prognostic factors. Men staged as having pN3 disease, defined as the presence of extranodal extension (ENE) in lymph node metastases or pelvic lymph node metastases, have the poorest prognosis, with reports of 5-yr penile cancer–specific survival (peCSS) as low as 0–20% [1], [2]. However, more recent studies reported considerably better 5-yr peCSS of 30–50% [3], [4].

National and international treatment guidelines recommend perioperative oncological treatment for men with adverse risk factors [1], [5]. However, the evidence supporting such recommendations is weak and contradictory [6], [7], [8], [9], [10], [11]. Swedish guidelines published in 2016 recommend treatment for a wider range of patients than before, namely for men with c/pN2–3M0 disease and to the subgroup of men with c/pN1M0 disease who have two ipsilateral inguinal lymph node metastases [12].

Of the approximately 150 men annually diagnosed with invasive penile cancer in Sweden, 10–20 meet the criteria for perioperative oncological treatment [13]. Since 2013, national tumour board meetings are held weekly. Furthermore, surgery for penile cancer has been centralised to two tertiary hospitals since 2015, while oncological treatment has not been centralised. In our earlier study on oncological treatment among Swedish men with penile cancer between 2000 and 2015, we found that fewer men than recommended received oncological treatment, although adherence to national guidelines was increasing [13].

The aim of this nationwide study was to assess the use of oncological therapy over two decades during which treatment recommendations were centralised and new national guidelines for enhanced perioperative treatment were introduced. Secondary aims were to compare survival between men who received perioperative oncological treatment and men who did not receive such treatment, and to identify reasons for the failure to administer oncological therapy despite the presence of indications according to guidelines.

## 2 Patients and methods

**Fig. 1:**
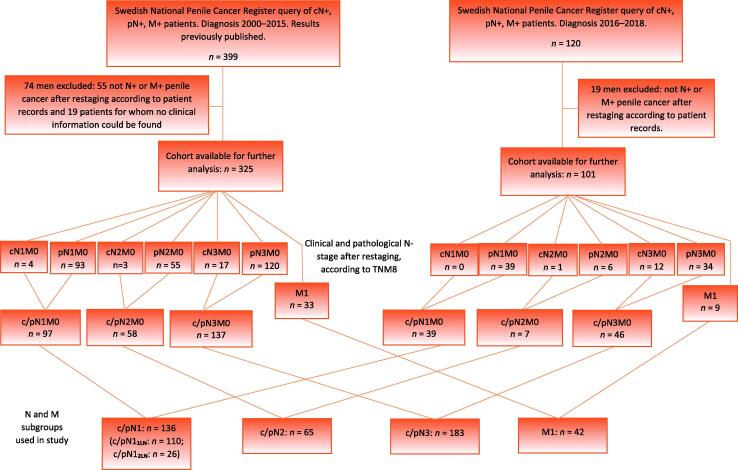
Flow chart showing the selection process for patients and subgroups.

Since 2000, virtually all Swedish men with penile cancer have been registered in the National Penile Cancer Register (NPECR) [14]. We used the NPECR to identify men with penile cancer metastasised to lymph nodes (clinical or pathological stage N1–3) or with distant metastases (stage M1) during 2016–2018. N and M stages according to the NPECR were only used for selection of the study cohort (Fig. 1). Medical records were retrieved. One reviewer collected clinical data, including detailed information on oncological treatments. For cases not receiving such treatments, even though indicated according to national guidelines, the reasons for omitting oncological therapy were registered.

A restaging of N and M status was conducted on the basis of information from the medical record review. No histopathology review was conducted. Clinical N stage was merged with pathological N stage to form five groups: c/pN1_1LN_M0, c/pN1_2LN_M0, c/pN2M0, c/pN3M0 and c/pN1–3M1, where *x*LN indicates *x* lymph nodes involved. Among men with M0 disease, 86% (79/92) had a pathologically confirmed N stage.

Patients were restaged according to both the Union for International Cancer Control) 7th and 8th editions of the TNM classification (TNM-7 and TNM-8) to allow comparisons with other published clinical materials [15]. The major change in N stage between TNM-7 and TNM-8 is that pN1 denotes one lymph node metastasis in TNM-7and one to two unilateral lymph node metastases in TNM-8. In order to be able to distinguish patients with one lymph node metastasis from those with two, we subgrouped c/pN1M0 into c/pN1_1LN_M0 and c/pN1_2LN_M0.

TNM-8 was used in all statistical analyses.

Of the 120 patients identified in the NPECR, 101 were confirmed as having stage c/pN1–3M0 or M1. Seventeen men who were found to have strictly localised disease and two who were diagnosed with other malignancies were excluded from further analyses.

Data on the cause of death were retrieved from the Swedish Cause of Death Register in December 2020. Penile cancer death was defined as penile cancer identified as the underlying cause of death.

To facilitate comparisons of treatments before and after updating of the national guidelines, we combined the patients in the present study with those from our earlier study of patients diagnosed up to 2015 [13]. The study population was updated on survival, cause of death, and TNM stage according to TNM-8 in December 2020. This provided treatment data for an overall cohort of 426 patients with penile cancer with lymph node or distant metastases reported in the NPECR for the period 2000–2018 for analysis.

Reasons for withholding oncological therapy in patients for whom such treatment was indicated were obtained from the medical records. Reasons were divided into five categories: (1) purportedly no indication; (2) patient’s request; (3) no information provided; (4) disease too advanced; and (5) frailty due to old age or comorbidity. The categories were merged into one group for men without contraindications (categories 1–3) and one group for men with contraindications to oncological treatment (categories 4 and 5). The former group thus represents patients theoretically eligible for oncological treatment.

The study was approved by the local ethics committee in Uppsala (Dnr 2015/520) and the Swedish Ethical Review Authority (Dnr 2020-00569).

### 2.1 Statistical analyses

Trend analysis of the use of curative treatment for men with an indication for perioperative oncological treatment over time was performed using a χ^2^ test for trends in proportions. peCSS was then calculated using Kaplan-Meier estimates. Patients were followed from diagnosis to death, emigration, or to the end of follow-up (December 31, 2020), whichever occurred first. Individuals who died of causes other than penile cancer were censored at their date of death. Cox proportional-hazard models were used to estimate hazard ratios (HRs) for penile cancer–specific mortality (peCSM) with 95% confidence intervals (CIs) in which time since penile cancer diagnosis was used as the underlying time scale. Four patients were not included in the Kaplan-Meier or Cox analyses because of missing information for the date of diagnosis. Therefore, 148 of 150 men with indication for treatment who did not receive treatment and 122 of 124 men with an indication for treatment who did receive treatment were included.

In the main analysis, mortality risks were compared between men receiving oncological treatment and men without such treatment. In a separate analysis, we further compared mortality risks between men who received oncological treatment and men who did not but did not have any apparent contraindications, reflecting a population that could possibly be targeted for oncological treatment. The updated recommendations in the national guidelines were introduced and in use from 2015, although not formally published until 2016. We therefore analysed men diagnosed before (2000–2014) and after (2015–2018) the new recommendations separately. We further assessed the influence of oncological treatment in men with more advanced (c/pN3M0) and less advanced (c/pN2M0+c/pN1_2LN_M0) penile cancer separately.

The multivariable models included stage, year of diagnosis, age at diagnosis, and curative perioperative oncological therapy. Schoenfeld residuals were used to verify the proportional hazards assumption.

R v4.0.3 was used for statistical analyses, *p* < 0.05 was considered statistically significant, and all tests were two-sided.

## 3 Results

### 3.1 Use of oncological treatment in penile cancer over two decades

Patient characteristics and treatments are presented in Table 1 for the entire study population during 2000–2018. Detailed treatment information for men diagnosed during 2016–2018 and for the entire study population (2000–2018) are presented in Table 2.

**Table 1:** Patient, disease, and treatment characteristics of 426 men diagnosed with N+ or M+ penile cancer by diagnosis period

Characteristic	Patients, *n* (%)			*p* value a
	2016–2018	2000–2015	Overall	
	(*n* = 101)	(*n* = 325)	(*n* = 426)	
Age at diagnosis				0.027
<40 yr	5 (5)	7 (2)	12 (3)	
40–59 yr	17 (17)	88 (27)	105 (25)	
60–79 yr	68 (67)	178 (55)	246 (58)	
≥80 yr	11 (11)	52 (16)	63 (15)	
T stage				0.033
Ta	–	4 (1)	4 (1)	
Tis	1 (1)	5 (2)	6 (1)	
Tx	–	19 (6)	19 (4)	
T0	–	1 (0)	1 (0)	
T1	17 (17)	53 (16)	70 (16)	
T2	37 (37)	129 (40)	166 (39)	
T3	38 (38)	83 (26)	121 (28)	
T4	8 (8)	21 (6)	29 (7)	
Data missing	–	10 (3)	10 (2)	
M stage (TNM-8)				0.849
M1	9 (9)	33 (10)	42 (10)	
M0	92 (91)	292 (90)	384 (90)	
**c/pN stage (TNM-8) for patients with M0 disease**				0.023
Total number of patients with M0	92	292	384	
c/pN1	39 (42)	97 (33)	136 (35)	
c/pN2	7 (8)	58 (20)	65 (17)	
c/pN3	46(50)	137 (47)	183 (48)	
**LNS in the curative setting**				0.125
Inguinal LNS, including sentinel node, extirpation, and inguinal LND (unilateral or bilateral)	80 (79)	264 (81)	344 (81)	
Pelvic LND (unilateral or bilateral)	34 (34)	160 (49)	194 (46)	
**CTx and RT treatment**				0.147
Any CTx and/or RT	56 (55)	172 (53)	228 (54)	
CTx and/or RT (including CRT)^2^ used at some point with curative intent	39 (39)	94 (29)	133 (31)	
CTx and/ or RT (including CRT) only used in the palliative setting	17 (17)	78 (24)	95 (22)	
RT and CRT				0.904
Any RT (including CRT)	34 (34)	120 (37)	154 (36)	
RT (including CRT) with curative intent b	15(15)	15 (5)	30 (7)	
RT in the palliative setting b	20 (20)	76 (23)	96 (23)	
CTx c				0.096
Any CTx	44 (44)	119 (37)	163 (38)	
CTx with curative intent b^,^^c^	33 (33)	65 (20)	98 (23)	
CTx in the palliative setting b	17 (17)	70 (22)	87 (20)	

CRT= chemoradiotherapy; CTx = chemotherapy; LND = lymph node dissection; LNS = lymph node surgery; RT = radiotherapy, TNM-8 = 8th edition of the Union for International Cancer Control TNM classification [15].

a Fisher’s exact test was used to test for possible differences in characteristics by year of diagnosis (2000–2015 vs 2016–2018).

b Patients who received treatment with curative intent and later in the palliative setting are reported in both groups.

c Neoadjuvant and adjuvant chemotherapy.

**Table 2:** CRT and RT treatment by N and M stage and treatment intent for patients diagnosed with penile cancer in 2016–2018 and in the overall cohort (2000–2018) a

	Patients diagnosed in 2000–2018, *n* (%)						Patients diagnosed in 2016–2018, *n* (%)					
Stage	c/pN1**_1LN_** M0	c/pN1**_2LN_** M0	c/pN2M0	c/pN3M0	N_any_M1	Total	c/pN1**_1LN_** M0	c/pN1**_2LN_** M0	c/pN2M0	c/pN3M0	N_any_M1	Total
Patients	110	26	65	183	42	426	30	9	7	46	9	101
Curative CRT	4 (4)	7 (27)	19 (29)	68 (37)	–	98 (23)	2 (7)	3 (33)	2 (29)	26 (57)	–	33 (33)
Palliative CRT	9 (8)	2 (8)	10 (15)	43 (23)	23 (55)	87 (20)	2 (7)	–	–	11 (24)	4 (44)	17 (17)
Curative RT	3 (3)	1 (4)	4 (6)	22 (12)	–	30 (7)	2 (7)	–	1 (14)	12 (26)	–	15 (15)
Palliative RT	18 (16)	2 (8)	11 (17)	49 (27)	16 (38)	96 (23)	3 (10)	1 (11)	1 (14)	11 (24)	4 (44)	20 (20)
Any CRT or RT with curative intent	9 (8)	8 (31)	26 (40)	90 (49)	–	133 (31)	3 (10)	3 (33)	3 (43)	30 (65)	–	39 (39)
Any CRT or RT, all settings	26 (24)	10 (38)	37 (57)	126 (69)	29 (69)	228 (54)	6 (20)	4 (44)	4 (57)	36 (78)	6 (67)	56 (55)

CRT = chemoradiotherapy; RT = radiotherapy.

a Some patients received more than one type of treatment.

Of the 101 men diagnosed during 2016–2018, 62 fulfilled the criteria for perioperative oncological treatment according to the national guidelines, of whom 36 (58%) received such treatment. Thirty-one men received chemotherapy, 30 at least one course of paclitaxel, ifosfamide and cisplatinum (PIC). Twenty-eight men received chemotherapy in a neoadjuvant setting and three men in an adjuvant setting. Thirteen men received adjuvant radiotherapy, 11 with fractionation as recommended in the national guidelines: 50 Gy at 2.0 Gy/fraction, or in the case of chemoradiotherapy (CRT), 50.4 Gy at 1.8 Gy/fraction. Eight men received both neoadjuvant PIC and adjuvant radiotherapy.

**Fig. 2:**
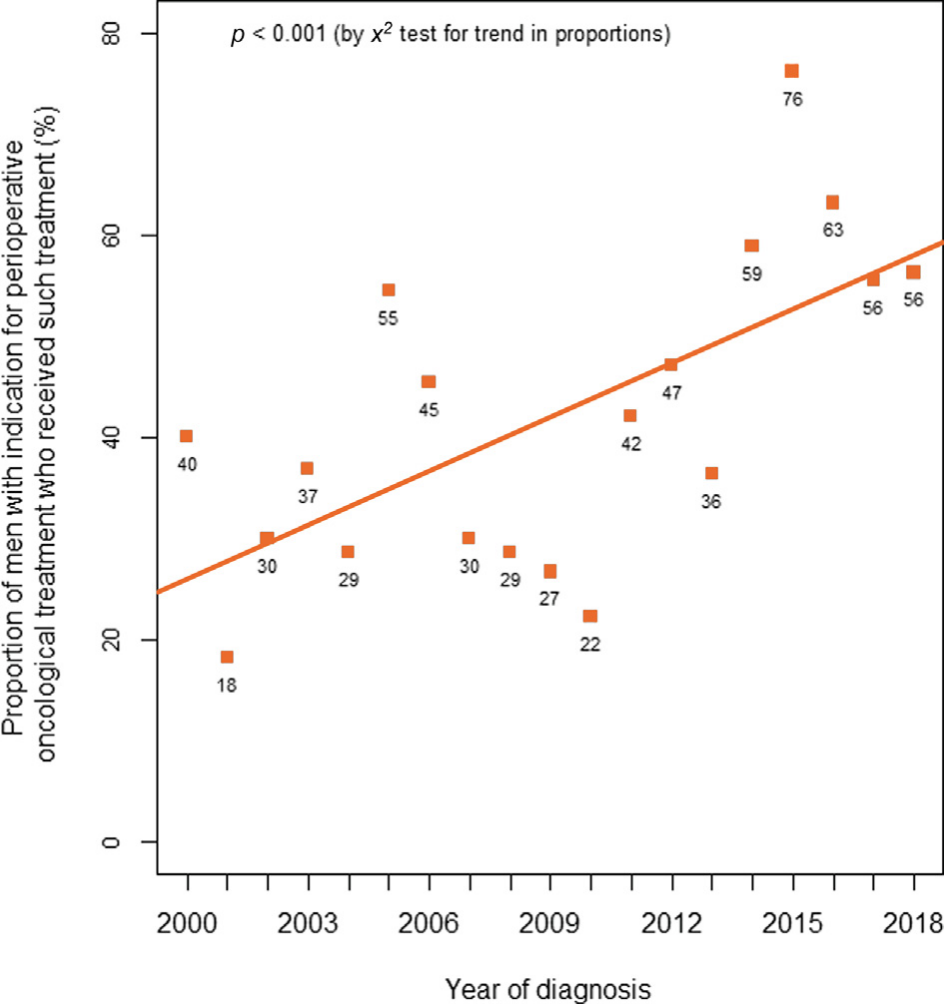
Annual trends in the use of perioperative oncological treatment for penile cancer. Each square represents the annual proportion of men with an indication for treatment who actually received it.

We observed a statistically significant increase in the proportion of patients receiving perioperative oncological therapy during the study period of 2000–2018 (Fig. 2). During the first 4 yr, 32% of patients with an indication for treatment received such treatment, which increased to 63% during the last 4 yr of the study period.

The group that received perioperative treatment had a lower median age and a larger proportion with c/pN3 disease in comparison to the group that did not receive such treatment (Supplementary Table 1).

### 3.2 Oncological treatment and survival over time

Of the 426 men who were diagnosed during the entire study period (2000–2018), 274 had an indication for perioperative oncological treatment. Of these, 124 received such treatment and 150 did not. The latter group included 59 men with contraindications: 30 were considered to have disease that was too advanced and 29 were considered too frail because of old age or comorbidity. Thus, 91 men without apparent contraindications could potentially have been treated.

**Fig. 3:**
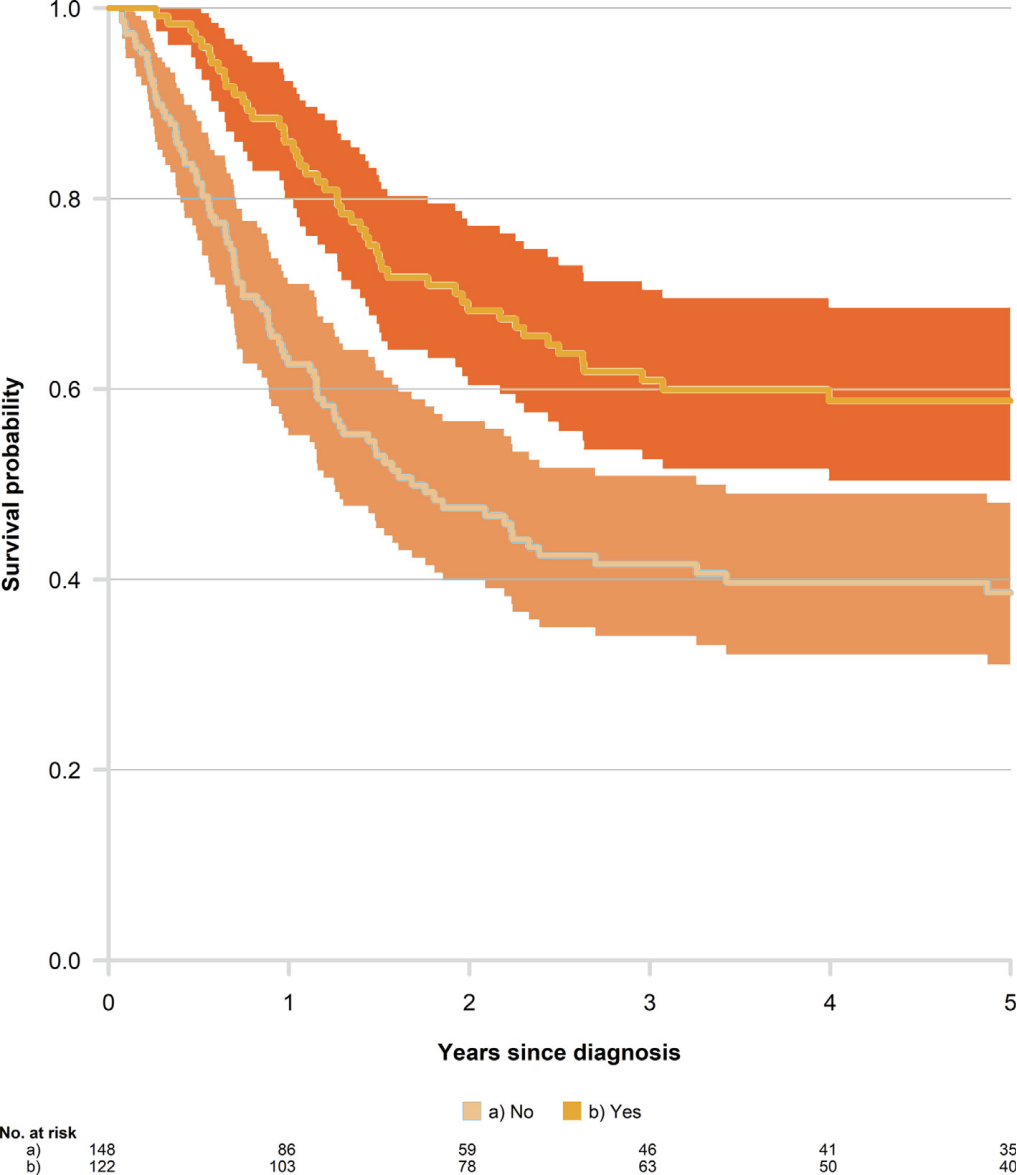
Kaplan-Meier curve for penile cancer–specific survival among patients with an indication for perioperative treatment stratified by receipt of treatment (yes vs no). Shaded areas present the 95% confidence intervals.

Overall, the Kaplan-Meier analysis suggests that peCSS was higher in the group receiving treatment than in the group not receiving treatment (Fig. 3).

Multivariable Cox regression analyses adjusted for stage, calendar period, and age showed that oncological treatment conferred a 59% reduction in peCSM (HR 0.41, 95% CI 0.28–0.61). More advanced stage (c/pN3M0 vs c/pN2M0+c/pN1_2LN_M0) was associated with higher mortality risk, while no associations were observed for age or time period (Table 3).

**Table 3:** Hazard ratios (HRs) a and 95% confidence intervals (CIs) for disease-specific death among Swedish men diagnosed with penile cancer between 2000 and 2018, with indication for perioperative oncological treatment

Variable	Patients, *n* (%)	HR (95% CI)
Oncological treatment		
No	148 (55)	Reference
Yes	122 (45)	0.41 (0.28–0.61)
Stage		
c/pN1_2LN_M0 or c/pN2M0	89 (33)	Reference
c/pN3M0	181 (67)	4.15 (2.66–6.49)
Year of diagnosis		
2000–2014	188 (70)	Reference
2015–2018	82 (30)	1.01 (0.67–1.53)
Age		
<65 yr	110 (41)	Reference
65–79 yr	121 (45)	1.00 (0.69–1.44)
≥80 yr	39 (14)	1.12 (0.65–1.93)

a Mutually adjusted for all covariates in the table.

Further analysis showed that the survival benefit for men who received oncological treatment remained even in comparison to men without contraindications, associated with a 37% reduction in peCSM (HR 0.63, 95% CI 0.40–0.98; Supplementary Fig. 1).

During 2015–2018, oncological treatment was associated with a 71% reduction in mortality risk (HR 0.29, 95% CI 0.09–0.96). By contrast, during 2000–2014, treatment was not clearly associated with mortality (HR 0.76, 95% CI 0.47–1.22; Supplementary Table 2).

Among patients with c/pN3M0 disease, oncological treatment was associated with a reduction in mortality risk (HR 0.58, 95% CI 0.35–0.97), while no clear association was observed among patients with c/pN2M0 or c/pN1_2LN_M0 disease (HR 0.70, 95% CI 0.26–1.89; Supplementary Table 3).

Of the nine men with M1 disease at diagnosis during 2016–2018, six men received palliative oncological therapy. Three men in this group received no oncological therapy and all died within 2 mo after diagnosis. One patient was alive at the end of follow-up; the remaining eight all had penile cancer assigned as their cause of death.

No treatment-related deaths were reported in the records and no patient experienced progression during oncological therapy to a stage that was inoperable following neoadjuvant treatment.

## 4 Discussion

In this nationwide study of men diagnosed with metastatic penile cancer in Sweden during 2000–2018, we observed an increase in perioperative oncological treatments over time. To the best of our knowledge, this is the first larger population-based study suggesting an improvement in survival with chemotherapy and radiotherapy in the perioperative setting.

The higher proportion of patients receiving oncological treatment during 2016–2018 in comparison to 2000–2015 in our previous study can be interpreted as an effect of the centralised tumour board recommendations. However, increasing use of oncological treatment has been described in other contemporary studies from centres with and without centralised penile cancer care [8], [16], [17], indicating that there may be additional explanations.

An increase in the use of perioperative chemotherapy has been reported in retrospective studies based on large databases in the USA, but these studies did not show any clear survival benefit associated with treatment [8], [16]. Other studies have reported high survival rates for patients who received perioperative treatment, but did not provide comparisons with survival for men without treatment. For example, a Danish study reported a 5-yr survival rate of 57% for 21 men with pN3 treated with perioperative radiotherapy [3]. Another population-based cohort has 51% cancer-specific survival at 5 yr in a group of 125 men with stage pN3 disease treated with adjuvant radiotherapy [17].

Cancer patients who receive oncological treatments often tend to be younger and healthier than patients not receiving such treatments, which, regardless of treatment, may explain the better survival in the treated group. However, while we found that patients in the treated group were indeed younger, they also had signs of more aggressive disease, indicated by a higher proportion of patients with stage c/pN3. We do not know why our results diverge from those of earlier studies, but different factors may have contributed. Restaging of TNM with better targeting of patients with an indication for treatment could be one reason. Furthermore, misclassification of stage could contribute to less efficient control of potential confounding, and any influence of residual confounding cannot be excluded. In addition, penile cancer is rare, so even large data sets are limited by low numbers, which could lead to chance findings.

We performed further analyses in which we compared men who received perioperative treatment to those who did not receive but were eligible for such treatment. To identify men with comorbidity, information was obtained from medical records. This approach has limitations, such as differences in record keeping and subjective assessment when collecting data. Still, it has the advantage of reflecting real-world decision-making. Penile cancer with lymph node metastases is an aggressive disease and 30 men had such rapidly progressing disease that even though they did not have distant metastases, curative treatment was deemed impossible. These patients have very poor prognosis that is not obvious from their disease stage.

The finding of better survival in the treated group remained in a comparison to the group of men who could have potentially been treated according to our model, lending further support to the benefit of oncological perioperative treatment.

In addition, the finding that a clear treatment benefit emerged during the later time period is in line with increasing use of standardised treatment over time.

The survival benefit observed for men with c/pN3 cancer but not for men with c/pN2 disease may be interpreted as supportive of a greater treatment gain for men with more advanced disease, with an overall higher risk of progression and death. However, as the group of men with c/pN2 disease was small, no firm conclusions can be drawn.

The finding that 23% (59/274) of men with an indication for treatment had contraindications is relevant when deciding on target levels for the proportion of patients in Sweden with an indication for treatment who should receive such treatment.

Consistently higher survival rates observed in contemporary studies, including our study, indicate that oncological therapy has an important role in the treatment of these patients. However, it is unclear which treatments are most efficacious. Comparisons of results for perioperative oncological treatment from different studies are fraught with uncertainty since there is considerable heterogeneity in patient selection and the types of surgery and oncological treatment. The randomised InPACT trial will answer some of the questions concerning optimal treatments and sequences for men with penile cancer and lymph node metastases [18], [19].

The strengths of our study include the population-based design, the long and essentially complete follow-up, and the large size. In addition to the above-mentioned limitations, the long follow-up over almost 20 yr involves important developments in clinical work-up that have certainly led to stage migration, which makes comparisons between different time periods somewhat uncertain. One example is the introduction of positron emission tomography/computed tomography, which has led to an increase in patients diagnosed with lymph node metastases. All cases in the study were restaged to improve comparability, but differences in clinical work-up cannot be compensated for in retrospect.

## 5 Conclusions

In conclusion, the use of perioperative oncological therapy in patients with penile cancer increased between 2000 and 2018. We further conclude that perioperative oncological treatment is associated with better survival in the more recent time period. However, evidence from randomised trials is warranted before conclusions regarding the potential benefits of perioperative oncological treatment can be drawn.

  ***Author contributions***: Emma Ulvskog had full access to all the data in the study and takes responsibility for the integrity of the data and the accuracy of the data analysis.

  *Study concept and design*: Ulvskog, Ahlgren.

*Acquisition of data*: Ulvskog.

*Analysis and interpretation of data*: Ulvskog, Persson, Fall, Kirrander, Ahlgren.

*Drafting of the manuscript*: Ulvskog, Persson.

*Critical revision of the manuscript for important intellectual content*: Fall, Kirrander, Ahlgren.

*Statistical analysis*: Persson, Fall.

*Obtaining funding*: Ulvskog.

*Administrative, technical, or material support*: None.

*Supervision*: Ahlgren.

*Other*: None.

  ***Financial disclosures:*** Emma Ulvskog certifies that all conflicts of interest, including specific financial interests and relationships and affiliations relevant to the subject matter or materials discussed in the manuscript (eg, employment/affiliation, grants or funding, consultancies, honoraria, stock ownership or options, expert testimony, royalties, or patents filed, received, or pending), are the following: None.

  ***Funding/Support and role of the sponsor*:** Funding for this work was obtained through the Agreement Concerning Research and Education of Doctors (ALF) from Örebro County and from the Research Committee of Örebro County. The sponsors had no role in data collection and management.

  ***Acknowledgments*:** The project was made possible by the Swedish Penile Cancer Register. The Regional Cancer Centre of Mid-Sweden contributed with statistical analyses.
