# Supplementary material for: Nationwide Data Support Centralised Decision-making in Penile Cancer Care: A Before-and-After Study on Guideline Adherence and Disease-specific survival for Patients with an Indication for Perioperative Oncological Treatment
Source: Eur Urol Open Sci. 2023 Mar 31;51:70–7. doi: 10.1016/j.euros.2023.03.005 (PMC10175737; doi:10.1016/j.euros.2023.03.005)

**Supplementary material**

**Supplementary Table 1 –** Men with indication for perioperative oncological treatment, 2000-2018: N-stage and age for patients who received such treatment and for patients who did not.

|  | Treatment | No treatment |
| --- | --- | --- |
| Total number of patients | 124 | 150 |
| Age at diagnosis, years  <40  40-59  60-79  ≥80  Mean age | 7 (6%)  37 (30%)  70 (56%)  10 (8%)  62.8 | 1 (1%)  36 (24%)  83 (55%)  30 (20%)  69.6 |
| N-stage  c/pN1_2LN_ M0  c/pN2M0  c/pN3M0 | 8 (6%)  26 (21%)  90 (73%) | 18 (12%)  39 (26%)  93 (62%) |

Supplementary Table 2 – Hazard ratios (HRs) and 95% confidence intervals (CIs) for disease-specific death among Swedish men diagnosed with penile cancer during 2000-2014 and 2015-2018, with indication for perioperative oncological treatment.

|  | 2000-2014 | | | 2015-2018 | | |
| --- | --- | --- | --- | --- | --- | --- |
|  |  |  |  |  |  |  |
|  | N | HR* | 95% CI | N | HR* | 95% CI |
| Oncological treatment |  |  |  |  |  |  |
| No, men without contraindications | 80 | Ref |  | 11 | Ref |  |
| No, men with contraindications | 37 | 2.54 | 1.50-4.29 | 20 | 2.41 | 0.79-7.37 |
| Yes | 71 | 0.76 | 0.47-1.22 | 51 | 0.29 | 0.09-0.96 |
|  |  |  |  |  |  |  |
| Stage |  |  |  |  |  |  |
| c/pN1_2LN_M0 + c/pN2M0 | 68 | Ref |  | 21 | Ref |  |
| c/pN3M0 | 120 | 3.4 | 2.06-5.62 | 61 | 7.80 | 2.59-23.44 |
|  |  |  |  |  |  |  |
| Age |  |  |  |  |  |  |
| <65 | 86 | Ref |  | 24 | Ref |  |
| 65-79 | 74 | 0.97 | 0.63-1.49 | 47 | 0.91 | 0.43-1.94 |
| 80+ | 28 | 1.34 | 0.70-2.55 | 11 | 0.61 | 0.21-1.81 |

*mutually adjusted for all covariates in the table

Supplementary Table 3 – Hazard ratios (HRs) and 95% confidence intervals (CIs) for disease-specific death among Swedish men diagnosed with penile cancer during 2000-2018, by tumor stage. Men with indication for perioperative oncological treatment.

|  | c/pN2M0+c/pN1_2LN_M0 | | | c/pN3M0 | | |
| --- | --- | --- | --- | --- | --- | --- |
|  |  | | |  | | |
|  | N | HR* | 95% CI | N | HR* | 95% CI |
| Oncological treatment |  |  |  |  |  |  |
| No, men without contraindications | 43 | Ref |  | 48 | Ref |  |
| No, men with contraindications | 13 | 3.12 | 1.04-9.43 | 44 | 2.49 | 1.50-4.12 |
| Yes | 33 | 0.70 | 0.26-1.89 | 89 | 0.58 | 0.35-0.97 |
|  |  |  |  |  |  |  |
| Year |  |  |  |  |  |  |
| 2000-2014 | 68 | Ref |  | 120 | Ref |  |
| 2015-2018 | 21 | 0.41 | 0.13-1.28 | 61 | 1.01 | 0.65-1.58 |
|  |  |  |  |  |  |  |
| Age |  |  |  |  |  |  |
| <65 | 41 | Ref |  | 69 | Ref |  |
| 65-79 | 35 | 1.76 | 0.64-4.89 | 86 | 0.83 | 0.56-1.24 |
| 80+ | 13 | 2.28 | 0.72-7.20 | 26 | 0.85 | 0.46-1.59 |

*mutually adjusted for all covariates in the table

Supplementary Figure 1 – Forest plots showing hazard ratios and 95% confidence intervals for the association between perioperative treatment and penile cancer specific survival in Swedish patients, 2000-2018. Multivariable models were controlled for stage, year of diagnosis and patient age at diagnosis. Perioperative treatment compared with no treatment in patients with or without contraindication to treatment.


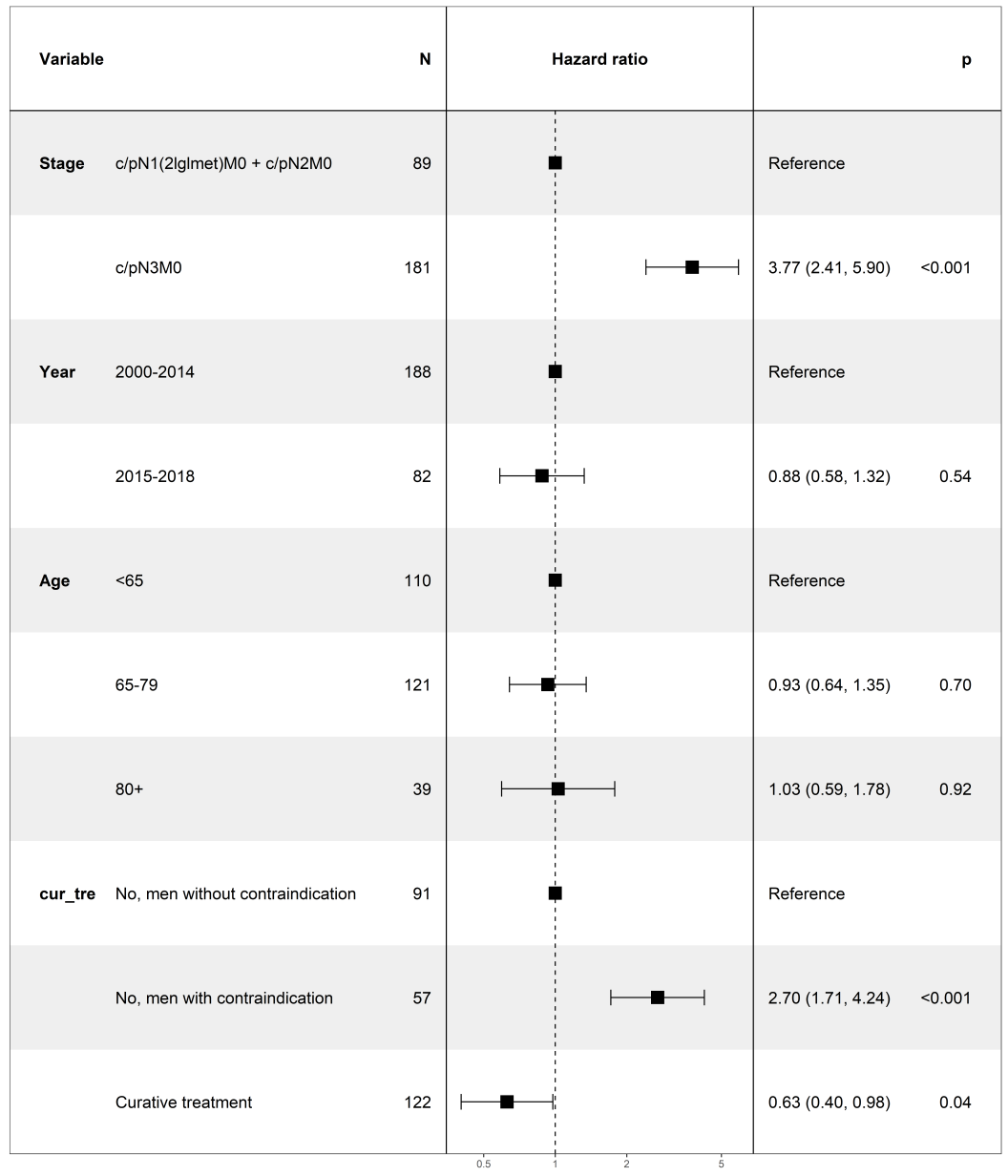

Supplement: Supplementary data 1 [file mmc1.docx]
